# Método rápido y preciso para la cuantificación de busulfán en muestras de plasma mediante cromatografía líquida acoplada a espectrometría de masas en tándem (LC-MS/MS)

**DOI:** 10.1515/almed-2022-0073

**Published:** 2022-08-12

**Authors:** Yolanda Villena-Ortiz, Laura Castellote-Bellés, Luisa Martinez-Sanchez, María I. Benítez-Carabante, Marta Miarons, Jaume Vima-Bofarull, Raquel Barquin-DelPino, Rosanna Paciucci, Francisco Rodríguez-Frías, Roser Ferrer-Costa, Ernesto Casis, Joan López-Hellín

**Affiliations:** Departamento de Bioquímica Clínica, Vall d’Hebron Hospital Universitari, Barcelona, España; Departament de Bioquimica i Biologia Molecular, Universitat Autonoma de Barcelona, Bellaterra, España; Departamento de Hematología y Oncología Pediátrica, Vall d’Hebron Hospital Universitari, Barcelona, España; Departamento de Farmacia Hospitalaria, Vall d’Hebron Hospital Universitari, Barcelona, España

**Keywords:** monitorización farmacoterapéutica, plataforma de espectrometría de masas, trasplante de células madre hematopoyéticas, validación analítica

## Abstract

**Objetivos:**

La administración de busulfán es ampliamente utilizada como parte del régimen de acondicionamiento en pacientes que se van a someter a trasplante de células madre hematopoyéticas. Se recomienda monitorizar las concentraciones plasmáticas de busulfán con el fin de optimizar la dosis en cada paciente y minimizar su toxicidad. El objetivo del presente estudio es validar un método analítico sencillo, rápido y costo-efectivo para la cuantificación de busulfán en plasma por cromatografía líquida acoplada a espectrometría de masas en tándem de aplicabilidad en la práctica clínica.

**Métodos:**

Las muestras de plasma se prepararon aplicando un protocolo de un solo paso de precipitación de proteínas. A continuación, las muestras se analizaron mediante elución isocrática en una columna C18. La fase móvil está constituida por 2 mM de acetato de amonio y ácido fórmico al 0,1% disuelto a una proporción 30:70 de metanol/agua. Como patrón interno, se utilizó busulfán-D_8_.

**Resultados:**

El tiempo total de medición se optimizó en 1,6 minutos. Las curvas de calibración fueron lineales entre 0,03 y 5 mg/L. El CV fue inferior al 8%. La exactitud de este método mostró un intervalo aceptable de entre 85 y 115%. No se observó interferencia por hemoglobina, lipemia o bilirrubina, ni siquiera a elevadas concentraciones de interferente. No se observó contaminación por arrastre ni efecto matriz al emplear este método. Se analizó el área bajo la curva de 15 pacientes pediátricos que recibieron tratamiento con busulfán previamente a un trasplante de células madre hematopoyéticas, y se estudió la correlación con las dosis administradas.

**Conclusiones:**

El método fue validado exitosamente y demostró ser suficientemente sólido para la monitorización farmacoterapéutica en un contexto clínico.

## Introducción

El busulfán (dimetanosulfonato de 1,4-butanodiol) es un antineoplásico perteneciente al grupo de los agentes bifuncionales alquilantes. Este fármaco induce la formación de aductos covalentes en el ADN provocando su ruptura y la inhibición de su replicación [1]. El busulfán se administra por vía intravenosa en pacientes con leucemia mieloide aguda y talasemia, como agente mieloablativo previo a un trasplante de células madre hematopoyéticas (TCH) [2], [3], [4]. Tal como ocurre con otros agentes quimioterápicos, el busulfán tiene un estrecho margen terapéutico y una elevada variabilidad interindividual en los parámetros farmacocinéticos [5, 6]. Para determinar la exposición sistémica máxima tolerada al busulfán, se utiliza el área bajo la curva (ABC) de la concentración plasmática en función del tiempo [7]. Un ABC elevada se asocia a un mayor riesgo de reacciones adversas tales como la enfermedad venooclusiva [8], [9], [10]. De este modo, se recomienda la monitorización de las concentraciones de busulfán en plasma con el fin de determinar la dosis correcta en un paciente y optimizar así la terapia, aumentando la eficacia y reduciendo su toxicidad [11].

Se han utilizado diversos métodos analíticos para cuantificar busulfán en plasma. Estos métodos se basan en el uso de técnicas como la cromatografía de gases acoplada a un detector de masas (GC-MS) [12, 13] y la cromatografía líquida de alta resolución (HPLC) acoplada a un detector ultravioleta (HPLC-UV) [14] o un detector de fluorescencia (HPLC-FLD) [15]. Y más recientemente, métodos por HPLC acoplada a espectrometría de masas en tándem (LC-MS/MS) [16], [17], [18], [19], [20], [21], [22], [23], [24], [25], [26], [27] (Tabla 1). Concretamente, la LC-MS/MS combina una elevada especifidad y sensibilidad analítica, con un coste menor y tiempos de respuesta significativamente menores, lo que simplifica el flujo de trabajo [28]. Sin embargo, las técnicas que se emplean actualmente para medir las concentraciones plasmáticas de busulfán son muy laboriosas y carecen de una evaluación detallada de interferentes endógenos o estabilidad a corto plazo. El propósito del presente estudio es validar un método analítico sencillo, rápido y costo-efectivo para la medida de busulfán en plasma de aplicabilidad en la práctica clínica.

**Tabla 1:** Diferencias entre nuestro método de LC-MS/MS y los métodos de LC-MS/MS publicados en los últimos 10 años para la cuantificación de Busulfán.

Referencia, (orden numérico)	Tiempo total de medición, min	Tipo de elución	Fase móvil	Columna analítica	Linealidad, mg/L	Estándar interno	Preparación de la muestra	Volumen de inyección, μL	Duración total del análisis^a^
Deng 2016 [17]	8	Gradiente	A: 20 mM acetato de amonio y 0,5% ácido fórmico en agua; B: 20 mM acetato de amonio y 0,5% ácido fórmico en metanol	Columna Supelcosil LC-18 (50 × 4,6 mm, 5 μm)	0,05–2,5	Busulfán-D_8_	Precipitación de proteínas	20	140
French 2014 [25]	4	Gradiente	A: 2 mM acetato de amonio y 0,1% ácido fórmico en agua; B: 2 mM acetato de amonio y 0,1% ácido fórmico en metanol	Columna Kinetex C18 (100 × 3 mm, 2,6 mm)	0,01–2	Busulfán-D_8_	Precipitación de proteínas y dilución	10	95
Jinjie 2020 [18]	5	Gradiente	A: 10 mM acetato de amonio y 0,1% ácido fórmico en acetonitrilo; B: acetonitrilo	Columna Hypersil Gold C18 (150 × 2,1 mm, 5 μm)	0,01–10	Busulfán-D_8_	Precipitación de proteínas	5	95
Matar 2020 [19]	2	Isocrática	Metanol/20 mM acetato de amonio en agua (90:10, v/v)	Columna Acquity UPLC BEH C18 (50 × 2,1 mm, 1,7 μm)	0,025–2	Busulfán-D_8_	Precipitación de proteínas y evaporación y reconstitución de la muestra	10	80
Moon 2014 [20]	10	Gradiente	A: 2 mM acetato de amonio y 0,1% ácido fórmico en agua; B: 2 mM acetato de amonio y 0,1% ácido fórmico en metanol	Columna XBridge™ C18 (100 × 2,1 mm, 3,5 μm)	0,025–5	Glipizida	Precipitación de proteínas	2	170
Nadella 2016 [21]	2	Isocrática	Acetonitrilo/10 mM formiato de amonio en agua (80:20, v/v)	Columna Kinetex C18 (50 × 2,1 mm, 2,6 μm)	0,04–2	Busulfán-D_8_	Precipitación de proteínas	10	50
Punt 2017 [26]	4,5	Gradiente	A: 0,1% ácido fórmico en agua; B: 0,1% acetato de amonio en acetonitrilo	Columna Acquity UPLC BEH C18 (50 × 2,1 mm, 1,7 μm)	0,01–10	Busulfán-D_8_	Precipitación de proteínas y dilución	3	105
Schofield 2019 [22]	8	Gradiente	A: 10 mM formiato de amonio en agua; B: metanol; C: acetonitrilo/2-propanol/acetona (6:3:1, v/v)	Columna Hypersil Gold C18 HPLC (50 × 3 mm, 5 μm)	0,01–5	Busulfán-D_8_	Precipitación de proteínas	25	140
Xiao 2018 [24]	3	Gradiente	A: 10 mM acetato de amonio y 0,1% ácido fórmico en agua; B: 0,1% ácido fórmico en acetonitrilo	Columna Luna C8 (2) HPLC (50 × 2 mm, 3 μm)	0,01–5	Busulfán-D_8_	Precipitación de proteínas y dilución	3	80
Actual	1,6	Isocrática	2 mM acetato de amonio y 0,1% ácido fórmico en metanol/agua (30:70,v/v)	Columna Mediterranean Sea 18 UPLC (50 × 2,1 mm, 1,8 µm)	0,03–5	Busulfán-D_8_	Precipitación de proteínas	2	45

^a^En la duración total del análisis se incluye la duración de la preparación de la muestra y del proceso analítico. Este tiempo es aproximado y se ha calculado a partir del pretratamiento descrito en cada manuscrito e incluyendo estándares, controles y cinco muestras.

## Materiales y métodos

### Reactivos

Se empleó busulfán, busulfán-D_8_, acetato de amonio de alta pureza, y bilirrubina de Sigma-Aldrich (St. Louis, MO, USA). La presentación comercial de busulfán-D_8_ fue de 100 μg/mL en metanol. Se empleó acetonitrilo de calidad HPLC/MS de Honeywell (Charlotte, North Carolina, USA) y metanol de calidad HPLC/MS de Fisher Scientific (Waltham, Massachusetts, USA). Se adquirió ácido fórmico de alta pureza de PanReac AppliChem (Barcelona, España). El reactivo Intralipid^®^ fue suministrado por Fresenius Kabi España (Barcelona, España). Se obtuvo plasma y plasma hemolizado de pacientes no tratados con busulfán.

### Equipamiento y condiciones

Las muestras de plasma se analizaron en el cromatógrafo líquido Nexera X2 acoplado a un espectrómetro de masas en tándem Shimadzu LCMS-8050 (Shimadzu, Kyoto, Japón), equipado con una fuente de ionización por electrospray. Los análisis cromatográficos se realizaron con la columna para cromatografía líquida de ultra alta resolución (UHPLC) Mediterranea Sea 18 1,8 µm de 50 × 2,1 mm de Teknokroma^®^. La columna se mantuvo a una temperatura de 40 °C. Los compuestos se eluyeron a una velocidad de flujo de 0,3 mL/min mediante elución isocrática de 1,6 minutos de duración. La fase móvil consistió en 2 mM de acetato de amonio y ácido fórmico al 0,1% disuelto en una proporción 30:70 de metanol/agua.

El detector de masas se configuró en modo ionización positiva. Se realizó el análisis en modo de monitoreo de reacciones seleccionadas (SRM) con una resolución de 0,5 *m*/*z*. En la Tabla 2 se muestran los parámetros de masa para el analito y el patrón interno. La temperatura de la interfaz se estableció en 300 °C, la temperatura del capilar de desolvatación se fijó en 250 °C, mientras que la temperatura del bloque térmico se fijó en 350 °C. Como gas nebulizador se empleó gas nitrógeno, a una velocidad de flujo de 3 L/min. También se empleó nitrógeno como gas de secado a una velocidad de flujo de 10 L/min. Se utilizó aire seco para calentar, a una velocidad de flujo de 10 L/min. El análisis de datos se realizó con el programa LabSolutions (Shimadzu).

**Tabla 2:** Ajustes del detector de espectrometría de masas para la transición seleccionada del analito y del estándar interno.

	Ión precursor (*m*/*z*) y fórmula lineal	Ión producto (*m*/*z*) y fórmula lineal	Voltajes Q1 y Q3, V	Energía de colisión, V	Tiempo de retención, min
Analito	264,1 [CH_3_SO_2_O(CH_2_)_4_OSO_2_CH_3_-NH_4_]^+^	151,0 [CH_3_SO_2_O(CH_2_)_4_-H]^+^	12/16	11	0,78
Estándar interno	272,1 [CH_3_SO_2_O(CD_2_)_4_OSO_2_CH_3_-NH_4_]^+^	159,0 [CH_3_SO_2_O(CD_2_)_4_-H]^+^	13/17	13	0,77

### Preparación de la muestra

#### Disoluciones madre, estándares de calibración y muestras de control

Se preparó una disolución madre de 500 mg/L de busulfán y otra de 500 mg/L de estándar interno en acetonitrilo (ACN) y se congelaron a −20 °C. Se preparó para cada curva de calibración un material de blanco, un calibrador cero, y seis calibradores con concentraciones de 5, 1, 0,5, 0,25, 0,08 y 0,03 mg/L de busulfán en plasma. Se prepararon cinco muestras de control de calidad (CC) con las siguientes concentraciones de busulfán en plasma: 4,0 mg/L (ALTA), 1,5 mg/L (MEDIA), 0,75 mg/L (BAJA), 0,09 mg/L (MUY BAJA) y 0,03 mg/L (límite inferior de cuantificación, LIC). La cantidad total de disolución madre añadida al plasma fue inferior al 5% del volumen final.

#### Pretratamiento de la muestra

La preparación de las muestras se realizó mediante precipitación de proteínas con ACN. Se mezcló una alícuota de 100 μL de plasma, estándar o control con 100 μL de disolución de trabajo de patrón interno (0,1 mg/L de busulfán-D_8_). A continuación se añadieron 600 µL de ACN. Las muestras se agitaron durante 1 minuto y finalmente se centrifugaron a 10.000*g* a temperatura ambiente durante 6 minutos. Seguidamente, se inyectaron 2 µL del sobrenadante en el sistema de LC–MS/MS.

### Linealidad

Para evaluar la linealidad del método, se analizaron las curvas de calibración una vez al día durante cinco días. Las curvas de calibración se obtuvieron dividiendo el área de pico del analito por el área de pico del estándar interno, y representando este valor frente a la concentración teórica del calibrador. Se permitió una desviación del 15% en todos los calibradores excepto en el LIC, que se fijó un máximo del 20%. Se confirmó la linealidad en el intervalo de medida cuando la recta de regresión lineal ponderada según la fórmula 1/x^2^ proporcionaba un coeficiente de correlación superior a 0,995 [29].

### Sensibilidad analítica

La evaluación del LIC se realizó como parte de la evaluación de la precisión y veracidad de la recta de calibración. El LIC se determinó como la concentración mínima de busulfán en plasma que se podía cuantificar con una desviación máxima del ±20% entre la concentración medida y la teórica, siguiendo las recomendaciones de la Administración de Alimentos y Medicamentos de los Estados Unidos (Food and Drug Administration, FDA) y la Agencia Europea del Medicamento (European Medicines Agency, EMA) [30, 31].

### Veracidad y precisión

Se realizó la evaluación de la veracidad y la precisión una vez al día durante tres días. Se analizaron cinco réplicas de cada muestra de CC (LIC, MUY BAJA, BAJA, MEDIA y ALTA) [31]. La desviación y el coeficiente de variación (CV) se calcularon a partir de los datos de cada serie por concentración. La incertidumbre de medida (IM) se calculó con la ecuación IM=1,96 × %CV. Los requisitos fijados fueron ±15% para el sesgo máximo permitido y ±15% para la imprecisión máxima permitida [30, 31].

Para medir el error de la medida de busulfán, se analizaron ocho muestras por duplicado del programa de garantía externa de la calidad de la Institución Holandesa para la Evaluación de la Calidad en los Laboratorios Clínicos (Stichting Kwaliteitsbewaking Medische Laboratoriumdiagnostiek, SKML) (intervalo de 0,71–3,47 mg/L). Se consideró aceptable si la diferencia obtenida era inferior al ±15%.

### Selectividad y arrastre

La selectividad de nuestro método se evaluó comparando las respuestas de las muestras de LIC con las respuestas de los blancos. Los blancos se prepararon a partir de 50 muestras de plasma sin busulfán. La interferencia por hemoglobina, lipemia y bilirrubina se evaluaron analizando muestras con una cantidad fija de busulfán (1,5 mg/L) en presencia de concentraciones crecientes de hemoglobina, Intralipid^®^ o bilirrubina. El cambio porcentual se calculó con la siguiente ecuación: %Interferencia=100*(C_I_ − C_0_)/C_0,_ donde C_0_ fue la muestra con busulfán en ausencia de interferente, y C_i_, la muestra con busulfán en presencia del interferente. Se consideró que una interferencia era significativa cuando el %Interferencia superaba el error total del busulfán a 1,5 mg/L (calculado como |sesgo%| + 1,96*CV%).

La contaminación por arrastre se analizó comparando el área de pico cromatográfico de un blanco inyectado inmediatamente después de una muestra de 4 mg/L, y el área de pico de un blanco inyectado inmediatamente después del estándar de 5 mg/L, con el área de pico del LIC. Las medidas se realizaron por duplicado durante 5 días. Se consideró aceptable una diferencia inferior al 5% [29].

### Efecto matriz, recuperación y eficiencia del proceso cromatográfico

El efecto matriz (EM), la recuperación (RE) y la eficiencia del proceso cromatográfico (EP) se evaluaron a dos concentraciones de busulfán (0,09 y 1,5 mg/L), aplicando el procedimiento descrito por Matuszewski [32]. El EM también se calculó a una concentración de busulfán de 4 mg/L. Se prepararon tres series de muestras para evaluar el EM, la RE y la EP: a) la serie 1 estaba compuesta por muestras con matriz de ACN; la serie 2 se realizó a partir de extractos del material de blanco y añadiendo el busulfán previamente al análisis; y c) la serie 3 estaba compuesta por muestras de plasma extraídas de forma ordinaria. El EM se determinó comparando la serie 2 con la serie 1, la RE comparando la serie 3 con la serie 2, y la EP comparando la serie 3 con la serie 1. Cada serie constó de seis mezclas de plasma independientes y se analizaron por triplicado.

La EM y la EP se consideraron aceptables si presentaban una desviación máxima ±15%. Para evaluar la RE, se valoró que los datos fueran consistentes, precisos y reproducibles.

### Estabilidad

Las muestras de CC BAJA y ALTA se dividieron en alícuotas de 0,1 mL y se analizaron inmediatamente después. Todos los análisis se realizaron por triplicado. Para determinar la estabilidad del busulfán a temperatura ambiente, las muestras de CC se analizaron tras 24, 48 y 72 horas (h) conservadas a temperatura ambiente [31]. Para analizar la estabilidad de la muestra refrigerada, se analizaron las muestras de CC a las 24 h, a las 72 h y a los 7 días conservadas a 4–8 °C. La estabilidad de las muestras congeladas a −20 °C se analizó a los 30 y 60 días. Para determinar la estabilidad tras los ciclos de congelación y descongelación, las muestras se descongelaron y se volvieron a congelar a −20 °C una vez al día durante 3 días, y se analizaron después de cada ciclo. Para evaluar la estabilidad de la muestra en el autoinyector, se conservaron los sobrenadantes en el autoinyector durante 24 h y durante 7 días para su posterior análisis.

Debido a la inestabilidad del busulfán previamente reportada, se realizó un estudio de estabilidad a corto plazo. Para ello se analizaron las muestras de CC conservadas tanto a temperatura ambiente como a 4–8 °C durante 2, 4, 6 y 8 h.

### Aplicación clínica

Todos los pacientes incluidos en el estudio fueron pacientes pediátricos a los que se administró busulfán por vía intravenosa cuatro días consecutivos durante 3 h, junto con fludarabina [33], fludarabina y tiotepa, ciclofosfamida [34] o ciclofosfamida y melfalán [35]. La dosis inicial administrada se basó en el ABC objetivo ajustada al peso corporal de cada paciente y siguiendo nomogramas de dosificación personalizados, según el procedimiento descrito en estudios anteriores [36]. Los nomogramas de dosificación se diseñaron para lograr un régimen de acondicionamiento mieloablativo o no mieloablativo. Se procedió a la depleción de células T *ex vivo* con timoglobulina o alemtuzumab en todos los pacientes que iban a someterse a un TCH por enfermedades no malignas y en aquellos pacientes con enfermedades malignas cuando el donante no era un familiar directo. Durante el tratamiento con busulfán no se administraron ni paracetamol ni azoles. Se administró levetiracetam como terapia profiláctica anticonvulsiva.

Para medir la concentración de busulfán en el plasma de los pacientes, se extrajeron muestras de sangre (n=75) en tubos vacutainer K_3_-EDTA (Becton Dickinson, Milan, Italia). A continuación, se trasportaron las muestras a temperatura ambiente, se centrifugaron a 2.500*g* durante 5 minutos a temperatura ambiente y se congelaron sin que hubiera transcurrido más de una hora desde que se recibieron en el laboratorio.

Para el cálculo del ABC, se extrajeron muestras de sangre inmediatamente antes de la infusión de quimioterápico (hora 0), al concluir la infusión, y 1, 2 y 3 h tras la infusión [36]. La dosis inicial administrada fue de entre 3 y 5 mg/kg/24 h de busulfán, independientemente de la edad (3 mg/kg/24 h en 3 pacientes; 4 mg/kg/24 h en 4 pacientes; y 5 mg/kg/24 h en 8 pacientes). A partir del día 2, la dosis de busulfán se ajustó a partir de los resultados del ABC real obtenido el día anterior.

El protocolo para la extracción, almacenamiento y análisis de sangre fue aprobado por el Comité Ético del Hospital Vall d’Hebron (EOM(AG)027/2021(5825)). Este estudio se realizó en conformidad con los principios de la Declaración de Helsinki y las leyes y regulaciones españolas y europeas.

## Resultados

El tiempo de retención medio fue de 0,78 minutos para el busulfán y de 0,77 minutos para el busulfán-D_8_. Se observaron formas de picos cromatográficos aceptables, que no presentaban ni colas ni interferencias (Figura Suplementaria 1).

### Linealidad

Todas las curvas de calibración realizadas en la fase de validación fueron lineales, en el intervalo de 0,03–5 mg/L (media de 0,9958) (Figura Suplementaria 2). Durante la fase de validación no se excluyó ningún calibrador.

### Sensibilidad analítica

El método permite cuantificar con precisión el busulfán a una concentración de 0,03 mg/L (n=40). La relación señal-ruido observada a una concentración de 0,03 mg/L fue 5 veces superior a la relación observada en el material de blanco.

### Veracidad y precisión

Tanto la imprecisión intraserial como interserial de las cinco muestras de CC fue <7,2 %CV (Tabla Suplementaria 1). El sesgo medio en todas las concentraciones fue inferior al 10% con respecto a la concentración nominal (n=15 para cada muestra de CC). Se estimó una incertidumbre de medida entre 7,25 y 12,6%.

Los resultados obtenidos en el programa de garantía externa de la calidad SKML demostró una buena concordancia con los valores asignados (n=16; y=0,99x – 0,5 mg/L; r^2^>0,97). Todos los resultados individuales obtenidos se mantuvieron dentro del rango de desviación permitida del ±15% con respecto a los valores consensuados para LC-MS/MS. La desviación media observada fue de +4,1%.

### Selectividad y arrastre

**Figura 1:**
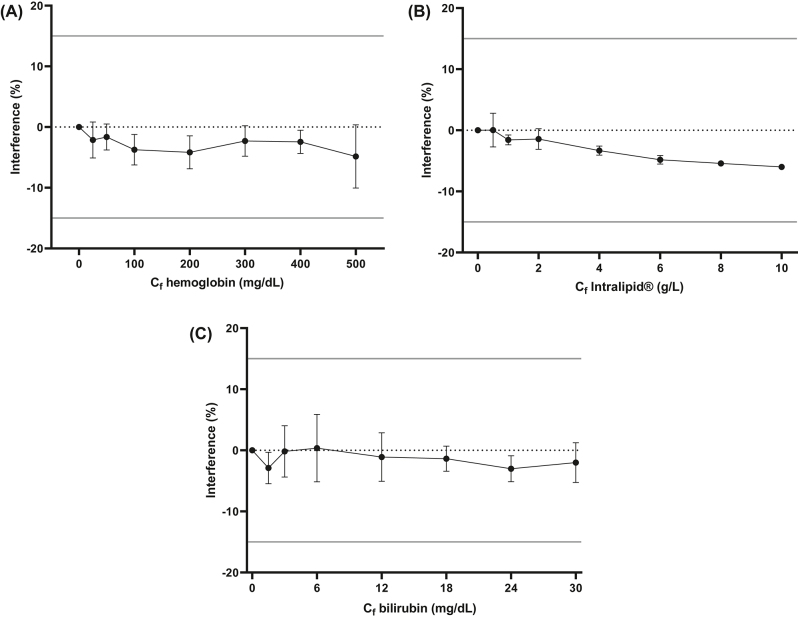
Interferogramas de hemoglobina (A), lipemia (B) y bilirrubina (C) a una concentración de busulfán de 1,5 mg/L (media y desviación estándar). C_f_ es la concentración del interferente en las soluciones: hemoglobina, Intralipid^®^ o bilirrubina.

En los materiales de blanco no se observaron interferencias en las transiciones analizadas (n=50). El método se validó con muestras de plasma que contenían una elevada concentración de hemoglobina, Intralipid^®^ o bilirrubina (Figura 1). El %Interferencia medio fue de −3,1%, −3,2% y −1,5%, respectivamente. El sesgo máximo calculado en todas las concentraciones de interferente fue inferior al error total del busulfán a 1,5 mg/L, 12,8%.

No se observó contaminación por arrastre por busulfán (n=10) ni por estándar interno (n=10) en el material de blanco.

### Efecto matriz, recuperación y eficiencia del proceso cromatográfico

Las tres pruebas arrojaron resultados consistentes para todas las concentraciones evaluadas (Tabla 3). El EM normalizado por el estándar interno fue del 92,9%, 100,2% y 99,8% para las concentraciones de busulfán de 0,09 mg/L, 1,5 mg/L y 4 mg/L, respectivamente. Las ratios de RE fueron del 94,6% y 103,3% para las concentraciones de 0,09 mg/L y 1,5 mg/L; las ratios de EP fueron del 98,2% y 97,1%, respectivamente.

**Tabla 3:** Resultados del estudio de eficiencia del proceso cromatográfico. Todos los resultados se expresan como porcentajes globales normalizados (media de los valores de factor matriz, recuperación o eficiencia del analito normalizados por el patrón interno).

Concentración de busulfán, mg/L	Efecto matriz, %				Recuperación, %				Eficiencia del proceso, %			
	n	Media, %	DE, %	CV, %	n	Media, %	DE, %	CV, %	n	Media, %	DE, %	CV, %
0,09	18	92,9	3,5	3,7	18	94,6	1,7	1,8	18	98,2	3,7	3,7
1,5	18	100,2	4,3	4,3	18	103,3	4,9	4,8	18	97,1	2,6	2,7
4	18	99,8	10,1	10,1	n.d.				n.d.			

n, tamaño de la muestra; DE, desviación estándar; CV, coeficiente de variación; n.d., no determinado.

### Estabilidad

**Figura 2:**
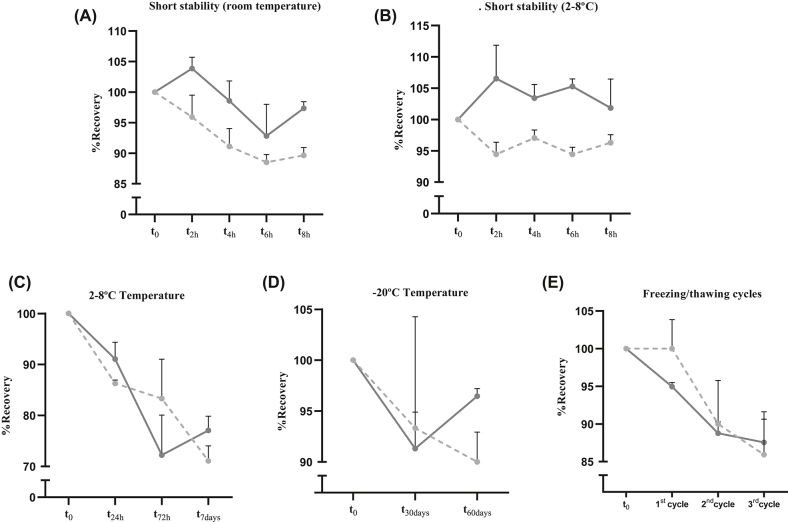
Estabilidad del busulfán en plasma conservado a temperatura ambiente (A), 2–8 °C (B, C) y −20 °C (D); y sometido a ciclos de congelación y descongelación (E). Las medias y desviaciones estándar se muestran por condición. La curva continua corresponde a la muestra de CC ALTA; la curva discontinua corresponde a la muestra de CC BAJA.

El busulfán es estable en plasma durante al menos 8 h a temperatura ambiente, 24 h a 2–8 °C, y hasta 2 meses a −20 °C (Figura 2). El estudio de estabilidad demostró que el busulfán no es estable cuando se conserva a temperatura ambiente durante 24, 48° 72 h (datos no mostrados). La concentración de busulfán disminuyó menos del 15% tras tres ciclos de congelación y descongelación (datos no mostrados). Los sobrenadantes de las muestras de busulfán en plasma permanecieron estables en el autoinyector durante 24 h: solo se observaron ligeras reducciones del 13,7 y del 8,9% a concentraciones de busulfán de 0,09 y 1,5 mg/L, respectivamente. En cambio, se observó una disminución más sustancial a los 7 días: 18,9% para la concentración de 0,09 mg/L, y 16,8% para la concentración de 1,5 mg/L de busulfán.

### Aplicación clínica

**Figura 3:**
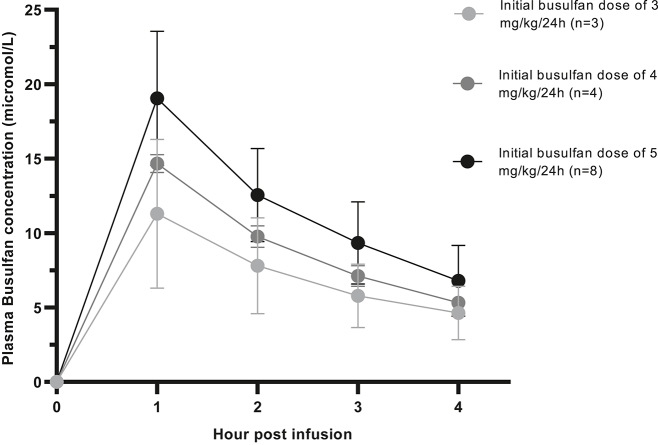
Distribución y variación de concentración de busulfán en plasma en muestras clínicas tras una dosis inicial de busulfán de 3 (n=3), 4 (n=4) y 5 (n=8) mg/kg/24 h.El punto central representa el valor medio, y los bigotes muestran la desviación estándar. 1 mg/L = 4,06 μmol/L.

Finalmente, se analizaron las concentraciones plasmáticas de busulfán en pacientes pediátricos (n=75 muestras; 7 niñas; edad media: 4,4 ± 3,5 años, rango de edad de 6 meses a 12 años). Las réplicas de las muestras confirmaron la fiabilidad de los resultados obtenidos (datos no mostrados). Los resultados de ABC correlacionaban adecuadamente con la dosis administrada (Figura 3). Tras las dosis iniciales de busulfán de 3 (n=3 pacientes), 4 (n=4 pacientes) y 5 (n=8 pacientes) mg/kg/24 h, las ABC variaban entre 931,8 y 1780,9, entre 1653,9 y 1795,4, y entre 1531,9 y 3230,0 µM min, respectivamente.

## Discusión

Presentamos un método analítico rápido, sencillo y robusto para monitorizar las concentraciones de busulfán en muestras de plasma mediante un analizador LC-MS/MS accesible para muchos laboratorios clínicos. El método descrito cumple los requisitos de veracidad y precisión y se ha validado para su aplicación en la práctica clínica.

Durante la monitorización farmacoterapéutica, es importante minimizar los tiempos de medición, ya que los resultados de los análisis deben ser enviados rápidamente, con el fin de que se puedan realizar cambios en el tratamiento, si fuese preciso. El método que presentamos presenta varias ventajas con respecto a otros métodos publicados para la monitorización de busulfán mediante LC-MS/MS [16], [17], [18], [19], [20], [21], [22], [23], [24], [25], [26], [27], entre los que se encuentran un menor tiempo de medición (Tabla 1). Los resultados se obtienen en menos de una hora y media. En este tiempo se incluyen el transporte y la recepción de la muestra, la preparación del analizador y de la muestra y el tiempo necesario para la verificación de los resultados. Con respecto a los procedimientos de preparación de la muestra citados en la Tabla 1, la duración total del tiempo de análisis se redujo a 45 minutos. Otra diferencia importante es que este método emplea un protocolo de elución isocrática con una única fase móvil. En consecuencia, dicho protocolo requiere un menor consumo de reactivos y simplifica el procesamiento del método. En comparación con otros métodos de elución isocrática para la cuantificación de busulfán [19, 21], nuestro intervalo de medición analítica es más amplio, reduciendo el número de muestras que es necesario diluir, lo que simplifica aún más la preparación de las muestras y el proceso analítico.

La mayoría de los errores de laboratorio se producen durante la fase preanalítica, siendo la interferencia analítica y la estabilidad del analito las principales causas. En un laboratorio clínico, es esencial tener en cuenta estas fuentes de error, con el fin de garantizar la obtención de resultados fiables, lo que garantiza nuestro método. Es importante destacar que en las publicaciones anteriores sobre métodos similares no se detallaban compuestos interferentes endógenos (Figura 1) ni se realizaron estudios de estabilidad a corto plazo (Figura 2).

Este estudio presenta algunas limitaciones. En primer lugar, se empleó una transición tanto para el busulfán como para el estándar interno y no se calculó la ratio entre dos transiciones para detectar posibles interferencias. En segundo lugar, relacionada con el estudio de contaminación por arrastre, no fueron incluidas concentraciones elevadas que se pueden encontrar en muestras de pacientes extraídas por vía intravenosa.

Este estudio está respaldado por profesionales de la Unidad de Oncohematología Pediátrica y el método se está implementando actualmente en nuestra unidad. Nuestro esfuerzo permitirá diseñar en el futuro estudios farmacocinéticos, de manera que se minimice la toxicidad, se aumente la eficacia de los tratamientos y se mejoren los resultados de los trasplantes llevados a cabo en nuestro centro.

## Supplementary Material

Supplementary Material

Supplementary Material

## Material Suplementario

La versión en línea de este artículo ofrece material suplementario (https://doi.org/10.1515/almed-2022-0073).
