# Supplementary figures and images for: Método rápido y preciso para la cuantificación de busulfán en muestras de plasma mediante cromatografía líquida acoplada a espectrometría de masas en tándem (LC-MS/MS)
Source: Adv Lab Med. 2022 Aug 12;3(3):272–81. [Article in Spanish] doi: 10.1515/almed-2022-0073 (PMC10197483; doi:10.1515/almed-2022-0073)

## Slide 1
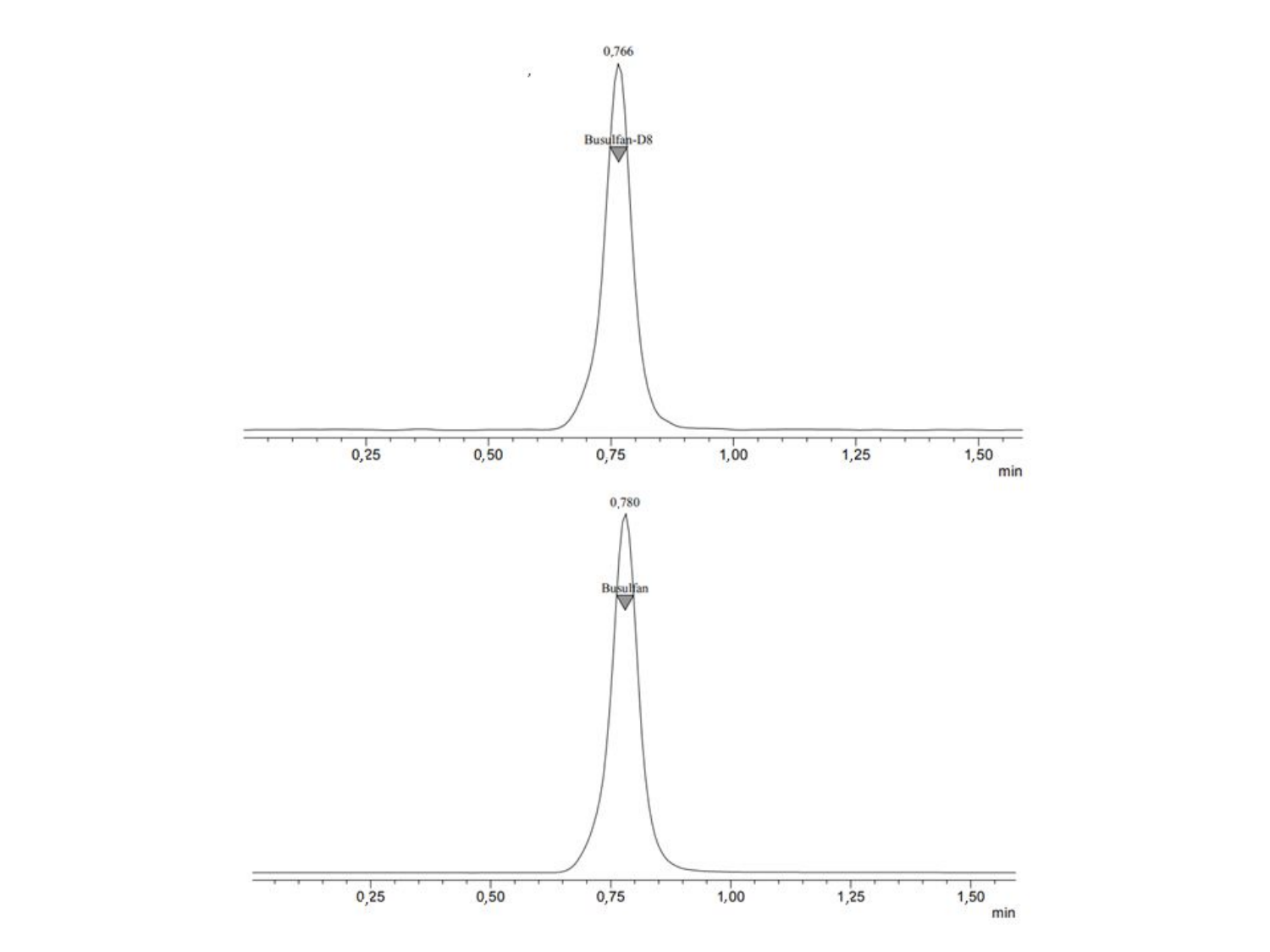

Supplement: Supplementary file 1 — Supplementary Material [file j_almed-2022-0073_suppl_001.pptx]

## Slide 1
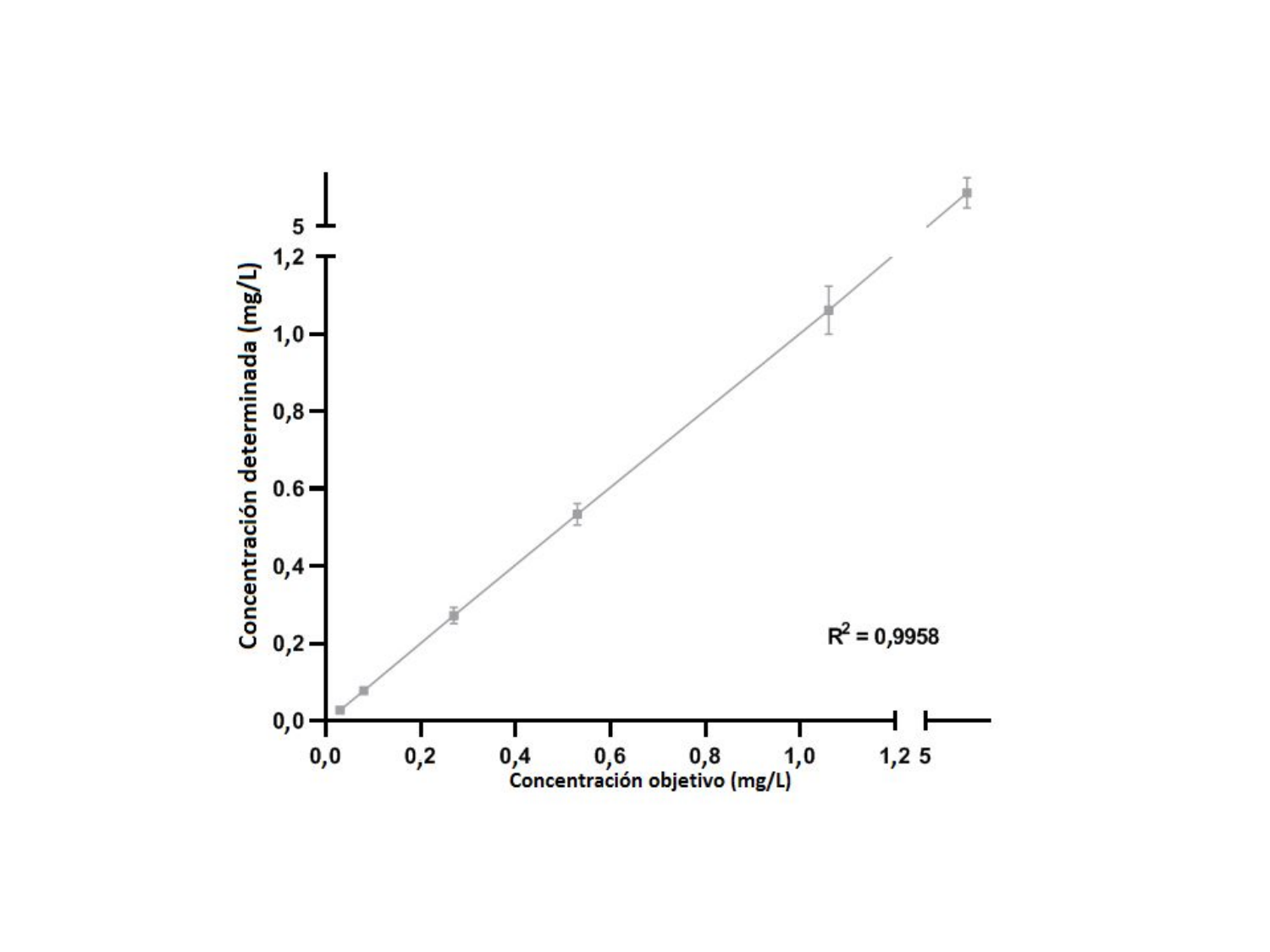

Supplement: Supplementary file 2 — Supplementary Material [file j_almed-2022-0073_suppl_002.pptx]
